# Schisandrin B Prevents Doxorubicin-Induced Chronic Cardiotoxicity and Enhances Its Anticancer Activity In Vivo

**DOI:** 10.1371/journal.pone.0028335

**Published:** 2011-12-02

**Authors:** Yang Xu, Zhen Liu, Jie Sun, Qiangrong Pan, Feifei Sun, Zhiyu Yan, Xun Hu

**Affiliations:** 1 Cancer Institute, Key Laboratory of Cancer Prevention & Intervention, National Ministry of Education, Key Laboratory of Molecular Biology in Medical Sciences, Zhejiang, China; 2 Department of Hematology, The Second Affiliated Hospital, Zhejiang University School of Medicine, Hangzhou, Zhejiang, China; Instituto Nacional de Câncer, Brazil

## Abstract

**Background:**

To mitigate the cardiotoxicity of anthracycline antibiotics without compromising their anticancer activities is still an issue to be solved. We previously demonstrated that schisandrin B (Sch B) could protect against doxorubicin (Dox)-induced acute cardiotoxicity via enhancing cardiomyocytic glutathione redox cycling that could attenuate oxidative stress generated from Dox. In this study, we attempted to prove if Sch B could also protect against Dox-induced chronic cardiotoxicity, a more clinically relevant issue, without compromising its anticancer activity.

**Methodology:**

Rat was given intragastrically either vehicle or Sch B (50 mg/kg) two hours prior to i.p. Dox (2.5 mg/kg) weekly over a 5-week period with a cumulative dose of Dox 12.5 mg/kg. At the 6th and 12th week after last dosing, rats were subjected to cardiac function measurement, and left ventricles were processed for histological and ultrastructural examination. Dox anticancer activity enhanced by Sch B was evaluated by growth inhibition of 4T1, a breast cancer cell line, and S180, a sarcoma cell line, in vitro and in vivo.

**Principal Findings:**

Pretreatment with Sch B significantly attenuated Dox-induced loss of cardiac function and damage of cardiomyocytic structure. Sch B substantially enhanced Dox cytotoxicities toward S180 in vitro and in vivo in mice, and increased Dox cytotoxcity against 4T1 in vitro. Although we did not observe this enhancement against the implanted 4T1 primary tumor, the spontaneous metastasis to lung was significantly reduced in combined treatment group than Dox alone group.

**Conclusion:**

Sch B is capable of protecting Dox-induced chronic cardiotoxicity and enhancing its anticancer activity. To the best of our knowledge, Sch B is the only molecule ever proved to function as a cardioprotective agent as well as a chemotherapeutic sensitizer, which is potentially applicable for cancer treatment.

## Introduction

Anthracycline antibiotics (doxorubicin, epirubicin, daunorubicin, idarubicin, etc.) are a class of anticancer agents whose roles in cancer chemotherapy are indispensable. Nevertheless, anthracycline treatment can be accompanied with an irreversible cardiotoxicity [1], [2], [3], [4] . Although it is still difficult to separate the primary mechanisms from the subsequent molecular events that lead to cardiotoxicity, many lines of evidence support that anthracycline antibiotics damage cardiomyocytes through generation of superoxide anion and further converting it to even more reactive hydroxyl free radical [1]. Anthracyclines can also disturb cellular antioxidative systems such as inactivating GSH peroxidase and exacerbating oxidative stress [5], [6]. Quenching ROS (superoxide anion, hydroxyl peroxide, and hydroxyl free radical) generated from Dox in cardiomyocytes is thus one of the major pharmacological approaches to prevent anthracycline-induced cardiotoxicity [1], [2], [3], [7], [8].

**Figure 1:**
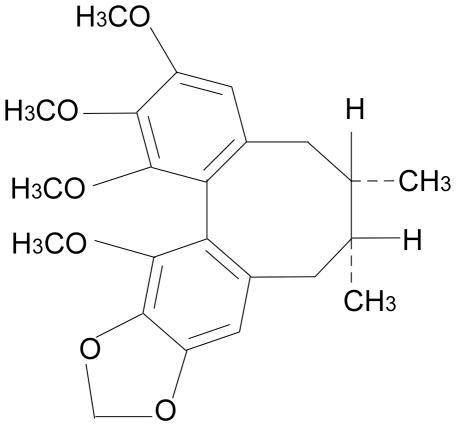
The chemical structure of schisandrin B.

Sch B (Fig. 1), the most abundant dibenzocyclooctadiene lignan present in Schisandra chinensis (Turcz.) Baill, can protect against oxidative stress including carbon tetrachloride-induced hapatotoxicity [9], myocardical ischemia/reperfusion injury [10], [11], and brain oxidative damage [12], through upregulation of GSH redox cycling. We recently demonstrated that Sch B was able to prevent Dox-induced acute cardiotoxicity, via enhancing cardiomyocytic glutathione redox cycling that could remove excessive ROS generated from Dox [13], [14]. Nevertheless, we do not know if this compound is also effective in preventing Dox-induced chronic toxicity. On the basis of previous reports, compounds that are effective in preventing acute cardiotoxicity (which appear within hours or days) induced by a singly high dose of Dox are not necessarily effective in preventing chronic toxicity (which takes weeks to appear) [15]. Thus, it is important to determine if Sch B can prevent anthracycline-induced chronic cardiotoxicity.

Another equally important issue is if Sch B could decrease Dox anticancer activity. We have demonstrated that Sch B was a dual inhibitor of P-glycoprotein and multidrug-resistance associated protein, and thus could sensitize drug-resistant cells to many structurally and functionally unrelated drugs including anthracycline antibiotics as the substrates of the 2 drug pumps [13], [16], [17], [18]. We also demonstrated that Sch B enhanced Dox anticancer activities in vivo against KBv200 (a human epidermoid carcinoma cell line overexpressing P-glycoprotein inoculated s.c. in nude mice) [19]. Fong et al. added the mechanism whereby the compounds in this chemical class inhibited P-glycoprotein [20]. Sch B was also able to enhance doxorubicin (vincristine, or mitoxantrone)-induced apoptosis in HMMC7721, a human hepatic cancer cell line, and MCF-7, a human breast cancer cell line, through activation of mitochondrial apoptotic pathway, without obvious enhanced toxicities toward normal cells, such as primary rat cardiomyocytes and primary human fibroblasts [16]. While the above studies were mostly performed in vitro, there is a need to determine if Sch B would interfere with Dox anticancer activities in vivo.

In this communication, we demonstrate that Sch B is able to protect against Dox-induced chronic cardiotoxicity, and enhances Dox anticancer activities against S180 sarcoma and 4T1 breast cancer in vivo.

## Results

### Sch B alleviates general toxicity of Dox

When compared with rats in control and Sch B groups that maintain similar growth rates, rats receiving Dox alone showed a much slower gain of the body weight starting from the 3rd week of treatment. Such Dox-related weight loss was partially alleviated in rats receiving SchB and Dox combined, which gained more weight than those receiving Dox alone, the difference was significant at the 4th week, *P*<0.05 (Figure S1A). Similar results were obtained in mice (Figure S1B). Moreover, 100 mg/kg of Sch B appeared to be more protective against weight loss than 50 mg/kg of Sch B, suggesting that its protective effect is dose-dependent. Regarding the mortality, one rat receiving Dox (n = 24) died of ascites six weeks after last dose of Dox, another 3 (n = 12) died of wasting or ascites, whereas only 1 receiving Sch B and Dox (n = 12) died of ascites between the 6th and 12th week after last dose of Dox. Taken together, these data demonstrate that Sch B alleviates general toxicity of Dox.

### Sch B prevents chronic myocardial damage

**Figure 2:**
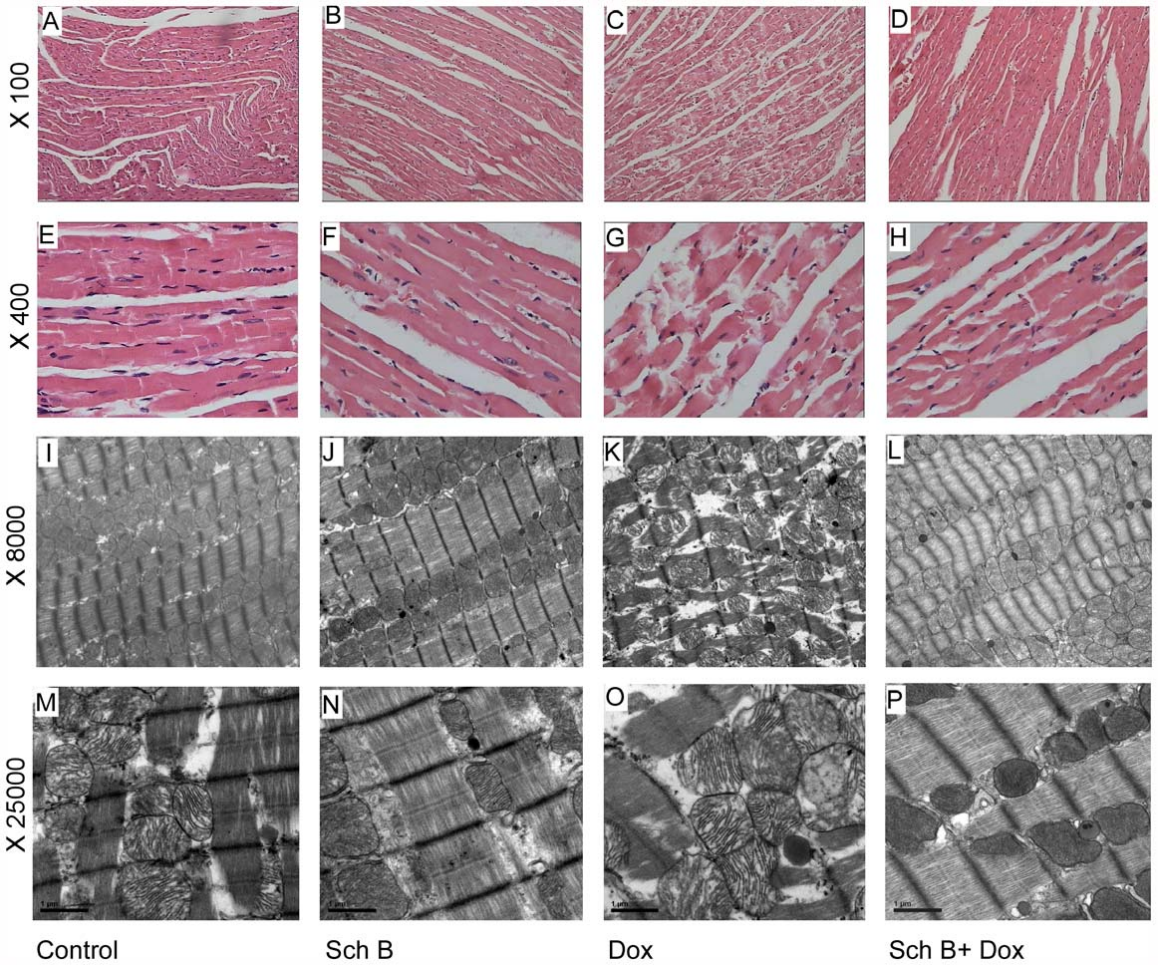
Sch B alleviates Dox-induced myocardial damage in left ventricles of rats. Rats were treated with Dox (2.5 mg/kg, i.p.) with or without pretreatment of Sch B, weekly over 5 weeks, followed by analysis of light or electron microscope 6 weeks after the last dose of Dox. Representative images of histology with hematoxylin-eosin staining (A–H) and ultrastructure (I–P) are shown.

To evaluate the myocardial damage induced by chronic Dox treatment, histological and ultrastructural changes of cardiomyocytes were examined. An overall view of the distribution of myocardial damage at the light microscopy level was shown in Figure 2A to 2H. No obvious abnormalities were observed in control (Figure 2A and 2E) and Sch B group (Figure 2B and 2F), while severe myocardial damages were found in Dox group, characterized by disorganization of myofibrillar arrays, cytoplasmic vacuolization (Figure 2C and 2G). Pretreatment with Sch B prevented marked cardiomyocytic vacuolization induced by Dox (Figure 2D and 2H). Similar pattern of damages was observed at the ultrastructural level (Figure 2I–2P). The control (Figure 2I and 2M) and Sch B groups (Figure 2J and 2N) did not show significant morphological abnormalities. However, Dox treatment led to loss of myofibril, vacuolization of cytoplasm (Figure 2K) and swelling of mitochondria with membrane disruption (Figure 2O), which was markedly attenuated by pretreatment of Sch B (Figure 2L and 2P).

### Sch B attenuates Dox-induced chronic cardiac dysfunction

**Figure 3:**
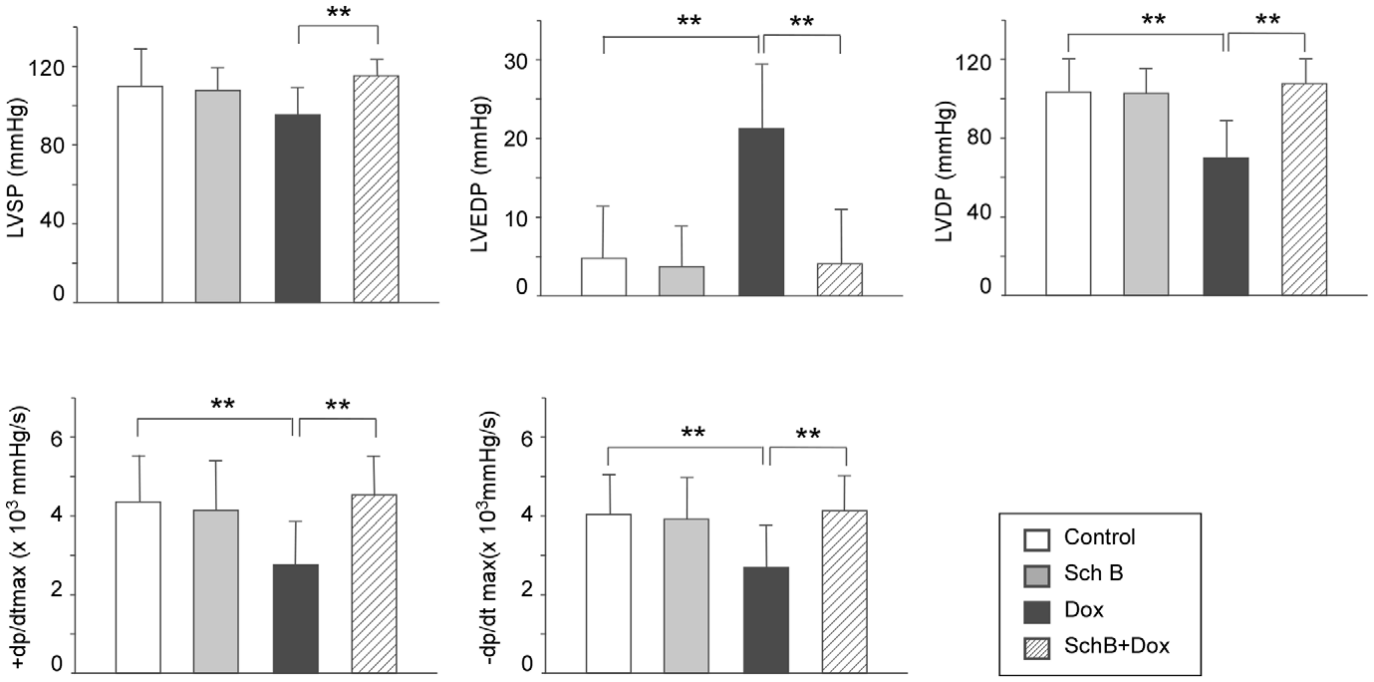
Sch B attenuates chronic cardiac functional loss caused by Dox. Rats were treated with Dox (2.5 mg/kg, i.p.) with or without pretreatment of Sch B (50 mg/kg intragastrically), weekly for 5 weeks. Cardiac function was measured in 6 weeks. **, P<0.05.

As shown in Figure 3, at the 6th week after last dosing of Dox, rats receiving Sch B alone did not show any obvious abnormal cardiac function compared with vehicle controls. Dox alone led to significant cardiac dysfunction characterized by decrease in LVSP, left ventricular developed pressure (LVDP), +dP/dt_max_ and −dP/dt_max_, as well as increased LVEDP. Pretreatment of Sch B prevented the Dox-induced loss of cardiac function. Similar results were obtained at the 12th week after last dosing of Dox (Figure S2). Taken together, these data indicated that Dox-induced chronic loss of cardiac function was significantly attenuated by pretreatment of Sch B.

### Sch B enhances Dox activities against tumor growth in vitro and in vivo

**Figure 4:**
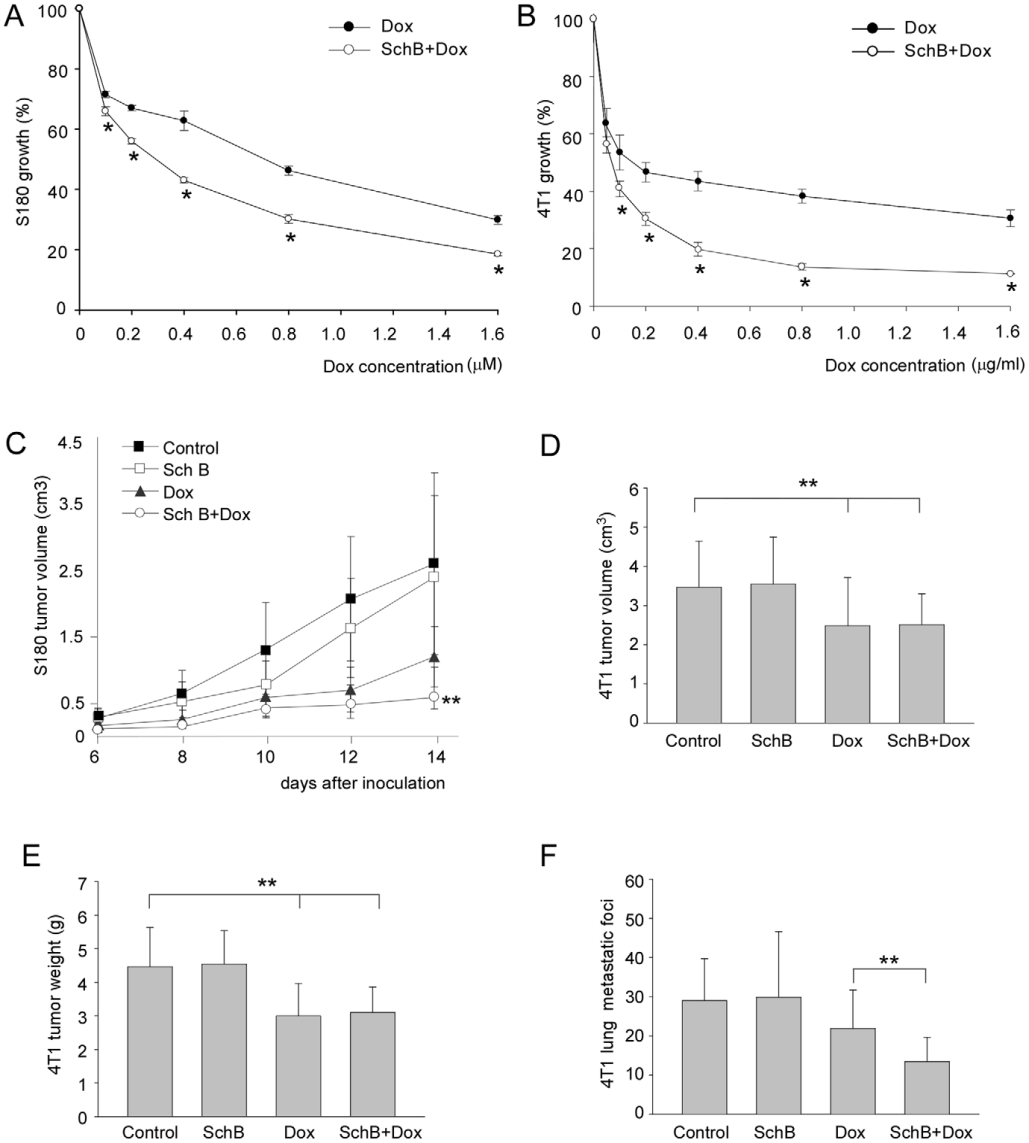
Sch B enhances the anticancer activity of the Dox in vitro and in vivo. A and B, in vitro growth inhibition rates of S180 cells (A) and 4T1 (B) by Dox in the presence or absence of Sch B. The inhibition rate was calculated as the ratio of the treated over the control. *, P<0.05. C–F, in vivo growth inhibition of S180 and 4T1 by Dox with or without Sch B. C, S180 tumor volume, **, P<0.05, Sch B+Dox versus Dox group; D, E, & F, 4T1 tumor volume, tumor weight, and pulmonary metastatic foci in 4T1 mice model (data were collected 28 days after 4T1 cell inoculation). The data were collected **, P<0.05, Dox, Sch B+Dox versus control group; E, 4T1 tumor weight, **, P<0.05, Dox, Sch B+Dox versus control group; F, , **, P<0.05, Sch B+Dox versus Dox group.

As shown in Figure 4, Sch B significantly increased Dox-induced growth inhibition of S180 in vitro (Figure 4A) and in vivo (Figure 4C). With 4T1 model, Sch B significantly enhanced Dox cytotoxicity toward 4T1 in vitro (Figure 4B). Although there was no significant difference of the implanted 4T1 tumor size and tumor weight between Dox+Sch B group and Dox alone group (Figure 4D and 4E), the combined treatment (Dox+Sch B) significantly reduced the spontaneous metastatic foci formed in lung (Figure 4F). We did not find metastasis in other organs, which was consistent with the general metastatic feature of this cell line, i.e., subcutaneous inoculation of 4T1 led to mainly pulmonary metastasis [21].

## Discussion

This communication, combined with our previous reports [13], [16], [17], [18], [22], [23], provides evidence that Sch B can protect Dox-induced acute and chronic cardiotoxicity and enhancing its anticancer activity.

In this study, slower weight gain was found in rats receiving Dox alone, while addition of Sch B could partially prevent weight loss caused by Dox. Decreased food intake due to gastrointestinal side effect and worsened general condition may contribute to slower growth of rats. Moreover, Dox has been shown to inhibit mTOR signaling and reduces cardiac mass [24]. mTOR plays a central role in regulating a variety of cellular processes, including cell growth, proliferation and energy metabolism. It is likely that Sch B partially relieve the Dox-induced mTOR inhibition in muscle or adipose tissue, thereby affecting body weight in rats. Development of ascites is at least in part due to chronic heart failure, there were less rats died of ascites in Sch B+Dox than Dox-only group (1 versus 3),which is consistent with cardioprotective function of Sch B.

It is difficult to remove ROS generated from Dox. First, the generation of ROS through quinone redox cycling is highly efficient, because it is an reaction catalyzed by a number of NAD(P)H-oxidoreductases [1]. Second, the molecular event is amplified due to its cycling nature, i.e., a molecule of Dox would continuously produce ROS as long as the cycle is not disrupted. Third, Dox, with a long in vivo elimination half-life, exerts chronic and chaotropic effects on cardiomyocytes [25], [26]. The reason some antioxidants like vitamin E and N-acetylcysteine are not effective in preventing chronic cardiotoxicity [15] is probably due to their pharmacokinetic features, e.g., their effectiveness is critically dependent on the cardiac distribution and elimination that should match Dox.

**Figure 5:**
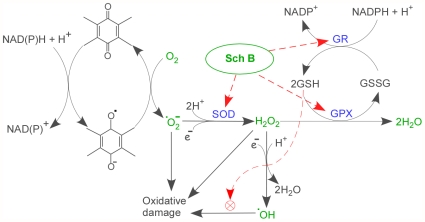
A proposed model for the protective role of Sch B in Dox-induced oxidative stress leading to cardiotoxicity. SOD, superoxide dismutase; GPX, glutathione peroxidase; GR, glutathione reductase ; ^•^O_2_^—^, superoxide anion; ^•^OH, hydroxyl radical; H_2_O_2_, hydrogen peroxide; ⊗,GSH directly scavenges hydroxyl radial through the reaction: GSH+R^•^→GS^•^+RH, then GS^•^ dimerizes to form GSSG.

In order to effectively remove ROS generated from Dox, it is essential to activate antioxidative systems which are a match to quinone redox cycling. Glutathione is the most abundant cellular antioxidant and glutathione redox cycling is the most effective cellular system against oxidative stress [27]. This redox cycle, in principle, has the capacity to cope with ROS generated from Dox. First, GSH redox cycling is enzymatically catalyzed and thus is highly efficient. Second, a molecule of GSH can quench many molecules of ROS due to its cycling nature. Third, as long as NADPH generation pathways via malic enzyme, cytosolic isocitrate dehydrogenase, and pentose phosphate pathway are not severely disturbed, GSH redox cycling will function properly and reduced GSH will remain a stable cellular pool at millimolar level, which is sufficient for reaction with ROS at nanomolar to micromolar range. While SOD converts superoxide anion (O^−^) to hydroperoxide, GSH redox cycling not only scavenges hydroxyl free radical, but also prevents excessive formation of hydroxyl free radical by converting superoxide anion and hydroperoxide to water and oxygen (Figure 5). We previously demonstrated that a single dosing of Sch B (50–100 mg/kg) was able to upregulate cardiac GSH redox cycling and superoxide dismutase (SOD) in rat and mice, and the increased activities of GSH redox cycling and SOD were lasted for at least 72 hours [13], which was particularly important for detoxification of continuous production of ROS from Dox, considering the long cardiac clearance of Dox, which was closely associated with chronic cardiac toxicity [25], [26].

The mechanism underlying Sch B-enhanced redox cycling remains poorly understood. Recently, Sch B was found to stimulate cytochrome 450-catalyzed NADPH oxidation reaction resulting in low level of ROS production, which in turn triggers redox signaling through ERK/Nrf2 pathway so as to promote cell survival [28], [29]. Yoshida et al. suggested that pitavastatin attenuates chronic doxorubicin cardiotoxicity through its antioxidant effect involving Rac1 inhibition [30]. Since Rac1 is a requisite component of NADPH oxidase, its role in Sch B-induced antioxidant activity remains to be studied.

In this study, we demonstrated that Sch B enhanced growth inhibition of S180 and 4T1 by Dox in vitro, consistent with our previous data [16]. We have shown that enhancement 0.6 or 1.2 nmol/kg (0.3–0.6 mg/kg)of Dox-induced apoptosis by Sch B in cancer cells was associated with the activation of caspase-9 rather than caspase-8 [16]. However, the signaling pathway upstream the caspase activation is not clear. Nishida et al. demonstrated that Sch B serves as a specific ATR protein kinase inhibitor and abolishes the G2/M and S-phase checkpoints in DNA damage response [31], interfering with effective DNA repair. Thus, disruption in ATR checkpoint pathway following Sch B treatment will sensitize cells to Dox-induced apoptosis. It seemed that Sch B had different effect on Dox in different tumors, i.e., Sch B enhanced Dox activity toward implanted S180 but not 4T1. This not surprising, because in vivo anticancer activity of a drug was to the great extent determined by the complex pharmacokinetic feature and the anticancer activities of a drug were often obvious in some animal models but not in others [32], [33]. The blood vessels in S180 tumors were drastically more pronounced and much more homogeneously distributed throughout the sections than those in 4T1 tumors (Figure S3). This could substantially limit the drug penetration and ultimately significantly reduce Dox and Sch B concentration in 4T1 tumor. Considering the dose-dependent characteristics (the least effective concentrations of both Dox and Sch B) [17], [18], [22], [23], the enhancing effect by Sch B could be abolished. Nevertheless, Sch B combined with Dox significantly reduced spontaneous metastatic foci in lung. This antimetastatic effect was conceivably resulted from killing metastatic cells at solitary cell stage (starting from metastatic cells separated from mother body to before forming a mass), similar to Sch B effect on Dox in vitro system where cancer cells were ‘solitary’ rather than tissue-like mass (tumor). Up to date, all the in vitro and in vivo studies without exception demonstrated that Sch B enhanced Dox anticancer activities [13], [16], [17], [18], [19], [20], [22], [34], [35], and there has been no single experiment in vitro and in vivo that ever demonstrated the adverse effect of Sch B on Dox anticancer activities (or any other tested anticancer agents, including anthracyclines, vinca alkaloids, taxanes, epipodophyllotoxins, antimetabolites, DNA topoisomerase inhibitors, tyrosine kinase inhibitors, among others) for the last 7 years of our continuous studies on this compound.

The mechanisms that Sch B has different effects on the susceptibility of cancer cells and cardiomyocytes toward Dox, as Dox basically induces apoptosis in both [1] have been discussed in great detail in our previous report [13], [16] and would not be discussed here.

To the best of our knowledge, Sch B is probably the only molecule so far reported to function as a cardioprotective agent against Dox as well as a chemotherapeutic sensitizer. This molecule has potential clinical application [13], [19].

## Materials and Methods

### Reagents

Dox was from Sigma, and Sch B (purity, 99.12% by HPLC) was from the Winherb Medical Science Co., Ltd, Shanghai, China.

### Cell lines

S180 and 4T1 cells were cultured in RMPI 1640 medium containing 10% fetal bovine serum in a humidified CO2 incubator at 37°C. Cell lines were obtained from and characterized by The Cell Bank of Type Culture Collection of Chinese Academy of Sciences according to the cell line authentication testing (vitality, species confirmation and interspecies contamination, DNA fingerprinting and mycoplasma contamination) and were used within 6 months after resuscitation.

### Animals

Male Sprague-Dawley rats, Balb/c mice were purchased from Shanghai SLAC Laboratory Animal Co., Ltd, Shanghai, China. Animals were housed in a controlled conditions of temperature (23±2°C), humidity (50±5%), and a 12 h light/dark cycle. The animals had free access to sterile food and water.

### Ethics

The study was approved by the institutional animal ethical committee of the hospital with approval IDzju2008-1-02-015.

### Cell proliferation assay

A nonradioactive cell proliferation assay (CellTiter96, Promega) was used to perform an MTT assay. S180 or 4T1 cells were seeded at a density of 5×10^3^ cells/well in 96-well plates and incubated with Dox of indicated concentration in the presence or absence of 50 µM Sch B as described by us previously [16], [17], [18], [22], [23]. At 72 hours after plating, MTT assays were performed according to the manufacturer's instructions. Data were collected by reading at 570 nm with a model ELX800 Micro Plate Reader (Bio-Tek Instruments Inc., Highland Park, USA). The percentage of growth inhibition was calculated by the following formula: percentage of cell inhibition = (mean absorbance in test wells)/(mean absorbance in control wells)×100.

### Chronic cardiotoxicity rat model

The protocol is modified based on the schedule described by Sacco G et al [36]. In brief, rats were randomly assigned into four groups (n = 24, per group): (a) control, (b) Sch B, (c) Dox, and (d) Sch B+Dox. Each rat, depending on the treatment group, was given intragastrically either vehicle (0.5% paraxamer) or Sch B (50 mg/kg) two hours prior to i.p. either saline or Dox (2.5 mg/kg) weekly over a 5-week period. Thus, cumulative dose of Dox were 12.5 mg/kg. Body weight was recorded weekly until the 6th week. At 6 and 12 weeks after last dose of Dox, 12 rats in each group were subject to hemodynamic measurement, respectively. At 6 weeks after last dose of Dox, left ventricles from 18 randomly selected rats (n = 4 for control and Sch B, n = 5 for Dox and Dox+Sch B, respectively) were removed, and each ventricle was split into two: one was fixed in 10% formaldehyde, and paraffin sections were stained with hematoxylin-eosin for histological examination; the other was processed for ultrastructural morphologic examination by electron microscopy, as described previously [37].

### Measurement of cardiac function

The studies were performed at the 6th and 12th week after last dosing of Dox. Left ventricular performance was analyzed in rat anesthetized with i.p. injections of chloral hydrate (360 mg/kg). A micro manometer-tipped catheter was inserted into the right carotid artery and advanced into the left ventricular under pressure control as described [38]. The left ventricular systolic pressure (LVSP), left ventricular end diastolic pressure (LVEDP), maximal rate of rise/fall left ventricle pressure development (+dP/dt_max_, −dP/dt_max_) were recorded. All pressure data were recorded on MedLab data acquisition system (Nanjing MedEase Co., Nanjing, China). The parameters described above were measured and recorded for at least 30 min.

### In vivo tumor models

Balb/c mice were inoculated subcutaneously with 2×10^6^ 4T1 cells/0.2 ml PBS or S180 cells/0.2 ml PBS. Within 24 hours, the mice were randomly assigned to four groups: (a) control, (b) Sch B, (c) Dox, and (d) Sch B+Dox. Each mouse received either vehicle or Sch B (100 mg/kg) intragastrically followed by saline or Dox (2.5 mg/kg) intraperitoneally every other day for a total of 7 doses. The size of tumor was recorded twice weekly. Mice were sacrificed when tumor sizes in control group were >2 cm^3^ (i.e. 28 days after cell inoculation), and the tumor and lungs were surgically removed for measurement.

### Statistical analysis

Data are expressed as mean ± S.E., Statistical comparisons between different groups were done by using one-way ANOVA followed by Tukey-Kramer multiple comparisons test (SPSS 10.0). Significance was accepted at p<0.05.

## Supporting Information

Figure S1 Effect of Sch B on the body weights of rats or mice receiving chronic Dox treatment. A, rats were assigned and treated as described in Methods. *, *P*<0.05, versus control or Sch B. B, Mice were inoculated with S180 cells and then assigned randomly into 5 groups: control group was given vehicle followed by saline, SchB group received Sch B and saline, Dox group received Dox only, SchB1+Dox group received 50 mg/kg Sch B followed by 2 mg/kg Dox, SchB2+Dox received Sch B 100 mg/kg followed by 2 mg/kg Dox. *, P<0.05, versus control or SchB; **, P<0.05, versus Sch B2+Dox.
(JPG)

Figure S2 Sch B attenuates chronic cardiac functional loss caused by Dox. Rats were treated with a Dox (2.5 mg/kg, i.p.) with or without pretreatment of Sch B (50 mg/kg intragastrically), weekly for 5 weeks. Cardiac function was measured in 12 weeks. A, LVSP, maximal left ventricle systolic pressure. B, LVEDP, maximal left ventricle end-diastolic pressure. C, LVDP, left ventricle developed pressure. +dP/dt, maximal slope of systolic pressure increment. E, −dP/dt, maximal slope of diastolic pressure decrement. **, P<0.05.
(JPG)

Figure S3 The microvasculature in S180 (A, B) xenograft tumor was drastically more than that in 4T1 tumor (C, D). The intratumoral microvasculature was detected by immunohistochemistry performed on paraffin embedded sections. Briefly, after deparaffination, sections were incubated in 0.3% H_2_O_2_ in TBS, and then blocked with 10% normal goat serum. The primary antibody anti-CD34 was applied for 1 h, rinsed in TBS, and followed by incubation with peroxidase labeled goat-anti-rat IgG antibody for 30 minutes. Sections were visualized by 3-amino-9-ethylcarbazole (AEC), counterstained with hematoxylin and mounted. Original magnifications: ×100.
(JPG)
