# Correction: Schisandrin B Prevents Doxorubicin-Induced Chronic Cardiotoxicity and Enhances Its Anticancer Activity In Vivo

**DOI:** 10.1371/annotation/7a1b01e4-b894-40ef-9c31-584e9c24ee0a

**Published:** 2012-01-23

**Authors:** Yang Xu, Zhen Liu, Jie Sun, Qiangrong Pan, Feifei Sun, Zhiyu Yan, Xun Hu

There is an error in the first affiliation. The correct first affiliation is: "1 Cancer Institute (Key Laboratory of Cancer Prevention & Intervention, National Ministry of Education, China; Key Laboratory of Molecular Biology in Medical Sciences, Zhejiang, China), the Second Affiliated Hospital, Zhejiang University School of Medicine" 

