# Supplementary figures and images for: Schisandrin B Prevents Doxorubicin-Induced Chronic Cardiotoxicity and Enhances Its Anticancer Activity In Vivo
Source: PLoS One. 2011 Dec 2;6(12):e28335. doi: 10.1371/journal.pone.0028335 (PMC3229562; doi:10.1371/journal.pone.0028335)

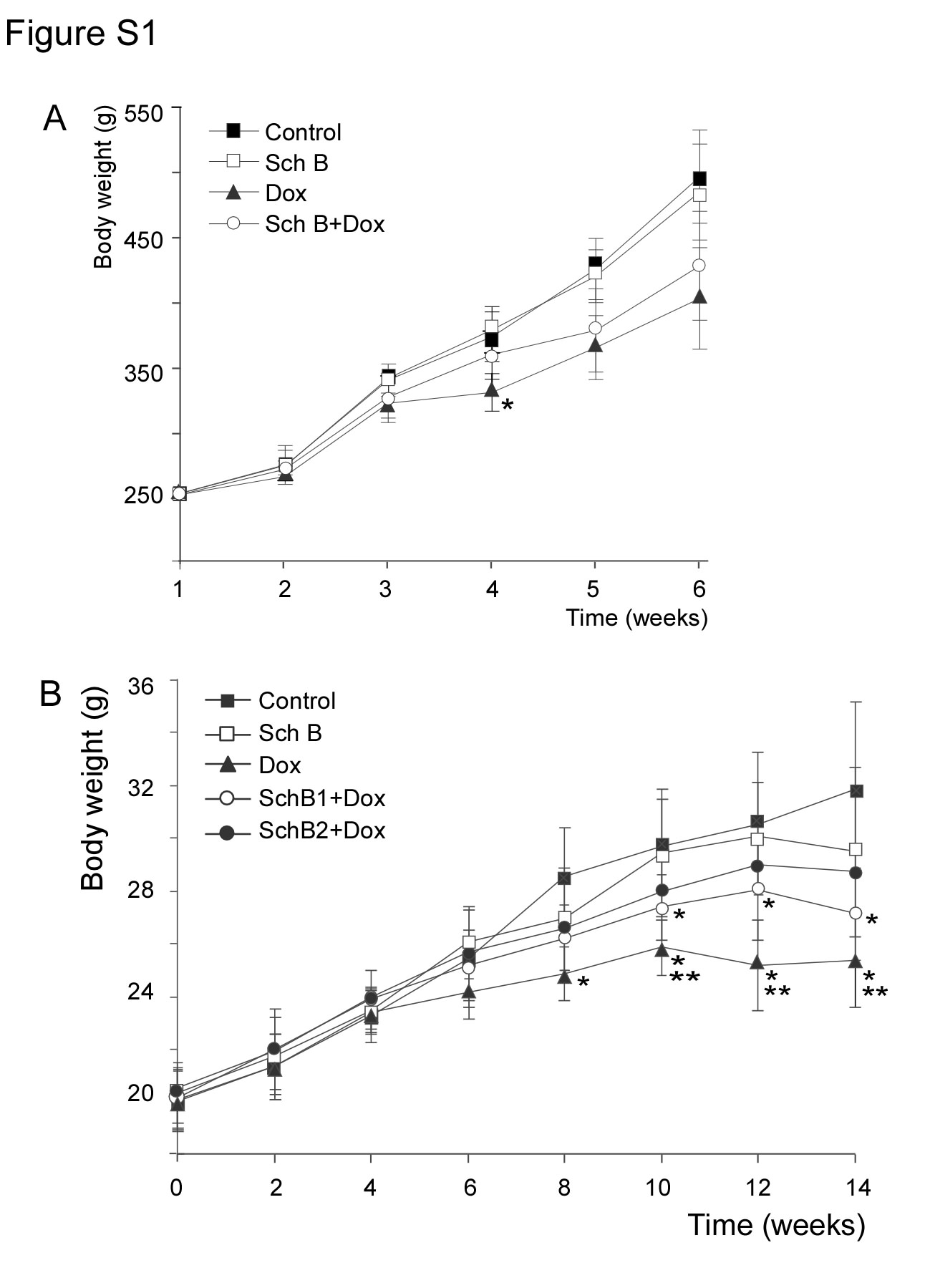

Supplement: Figure S1 — Effect of Sch B on the body weights of rats or mice receiving chronic Dox treatment. A, rats were assigned and treated as described in Methods. *, P<0.05, versus control or Sch B. B, Mice were inoculated with S180 cells and then assigned randomly into 5 groups: control group was given vehicle followed by saline, SchB group received Sch B and saline, Dox group received Dox only, SchB1+Dox group received 50 mg/kg Sch B followed by 2 mg/kg Dox, SchB2+Dox received Sch B 100 mg/kg followed by 2 mg/kg Dox. *, P<0.05, versus control or SchB; **, P<0.05, versus Sch B2+Dox. (JPG) [file pone.0028335.s001.jpg]

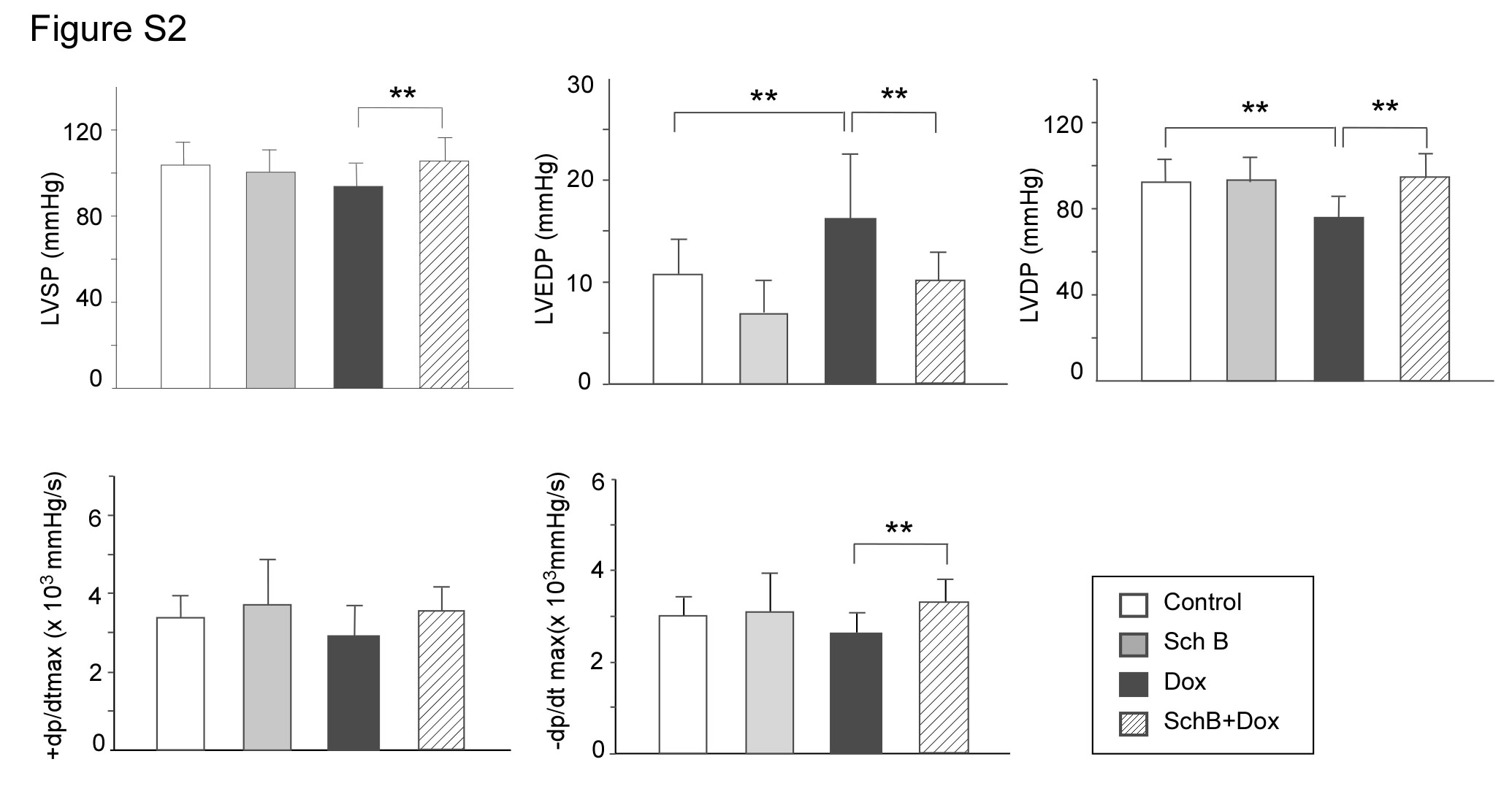

Supplement: Figure S2 — Sch B attenuates chronic cardiac functional loss caused by Dox. Rats were treated with a Dox (2.5 mg/kg, i.p.) with or without pretreatment of Sch B (50 mg/kg intragastrically), weekly for 5 weeks. Cardiac function was measured in 12 weeks. A, LVSP, maximal left ventricle systolic pressure. B, LVEDP, maximal left ventricle end-diastolic pressure. C, LVDP, left ventricle developed pressure. +dP/dt, maximal slope of systolic pressure increment. E, −dP/dt, maximal slope of diastolic pressure decrement. **, P<0.05. (JPG) [file pone.0028335.s002.jpg]

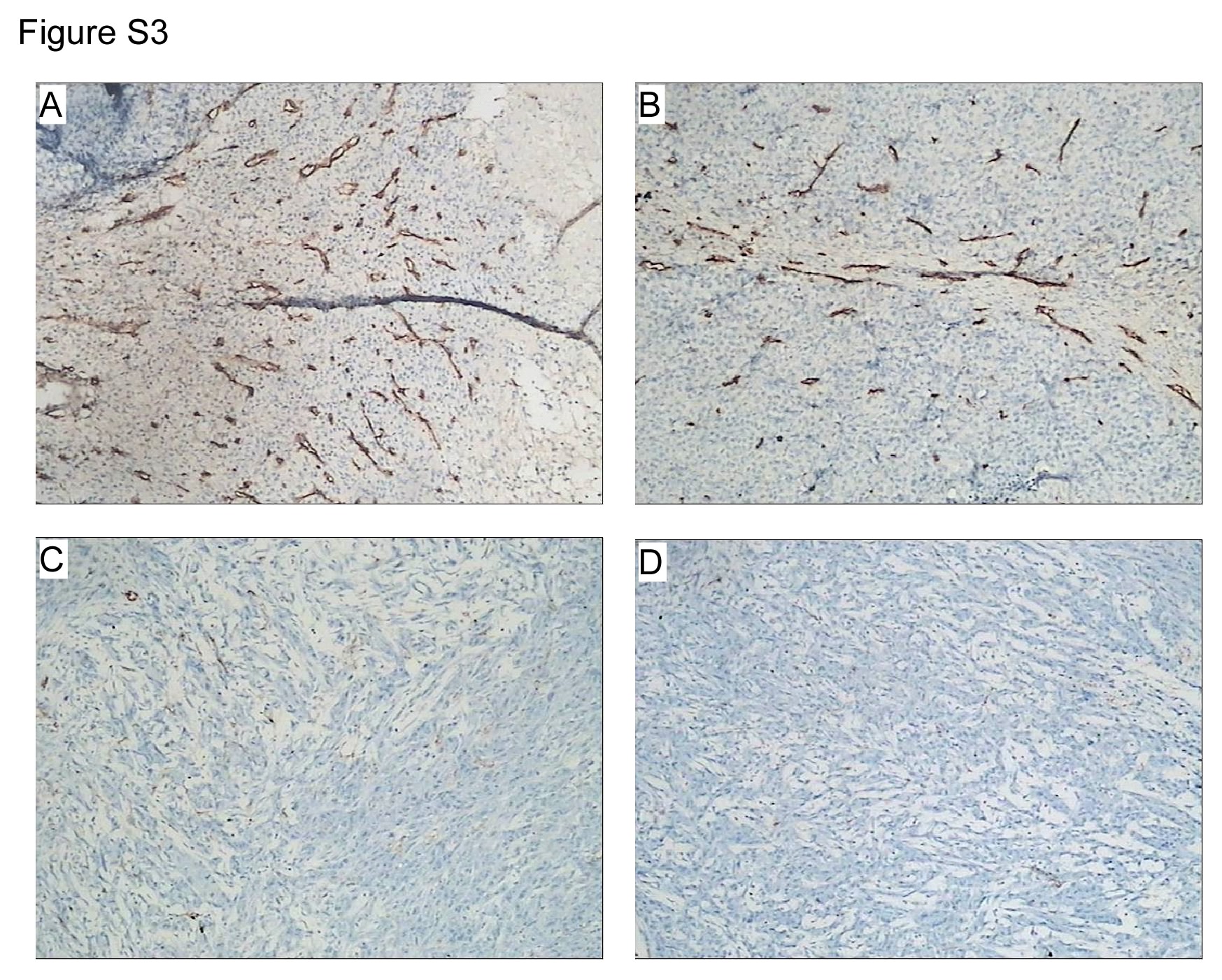

Supplement: Figure S3 — The microvasculature in S180 (A, B) xenograft tumor was drastically more than that in 4T1 tumor (C, D). The intratumoral microvasculature was detected by immunohistochemistry performed on paraffin embedded sections. Briefly, after deparaffination, sections were incubated in 0.3% H2O2 in TBS, and then blocked with 10% normal goat serum. The primary antibody anti-CD34 was applied for 1 h, rinsed in TBS, and followed by incubation with peroxidase labeled goat-anti-rat IgG antibody for 30 minutes. Sections were visualized by 3-amino-9-ethylcarbazole (AEC), counterstained with hematoxylin and mounted. Original magnifications: ×100. (JPG) [file pone.0028335.s003.jpg]
